# Supplementary material for: Determining the Relationship of Meteorological Factors and Severe Pediatric Respiratory Syncytial Virus (RSV) Infection in Central Peninsular Malaysia
Source: Int J Environ Res Public Health. 2023 Jan 19;20(3):1848. doi: 10.3390/ijerph20031848 (PMC9914795; doi:10.3390/ijerph20031848)
Supplement: Supplementary file 1 [file ijerph-20-01848-s001.zip › ijerph-2103650-supplementary.pdf]

Supplementary analysis 1: To support the significance of Spearman Rank Correlation by testing with a different categorical variable (Age less than 2 years)

Table S1. Correlation of meteorological factors with RSV cases

Meteorological factors	Range	Mean±S.d	Correlation coefficient[a]	p-value
Rainfall (mm)	72-678	308.5±137.5	-0.014	0.436
Rainy Days (n)	9-29	17.9±4.7	-0.051	0.006**
Temperature (°C)	26.7-29.4	28.4±0.6	0.040	0.029*
Relative Humidity (%)	62.9-82.3	72.7±3.9	-0.030	0.106*

[a] indicates results from Spearman Rank Correlation
*significance level at $p<0.05$
** significance level at $p<0.01$

Results: From this correlation model, we can see similar strength of correlation between children aged less than 2 years and RSV status. The only significance results were seen in temperature and rainy days, which have the same strength of correlation factor with RSV status model. Even though rainfall and relative humidity were not significant, the direction of correlation are similar with close approximation of correlation factors between the 2 models.

Supplementary analysis 2: Yearly analysis of 2 peaks

(a) First monsoon season (April to August)

Table S2. Correlation of 1st monsoon season meteorological factors with RSV cases

Compared to our yearly baseline results, stronger significant correlation factors are seen in rainfall (-0.102), relative humidity (-0.147) and rainy days (-0.139) with p value<0.001. Temperature reported weaker correlation factor and it is not significant.

Meteorological factors	Range	Mean±S.d	Correlation coefficient[a]	p-value	B-value[b]	p-value
Rainfall (mm)	72-598	279.2±129.7	-0.102	0.001**	-0.000	0.002**
Rainy Days (n)	9-23	16.0±4.3	-0.139	0.001**	-0.011	0.001**
Temperature (°C)	27.0-29.3	28.8±0.4	0.014	0.645	0.03	0.216
Relative Humidity (%)	64.7-82.3	72.4±3.4	-0.147	0.001**	-0.013	0.001**

[a] indicates results from Spearman's rank correlation
[b] indicates results from multiple bivariate logistic regression
** significance level at $p<0.01$

(b) Second Monsoon Season (October to March)

Table S3. Correlation of 2nd monsoon season meteorological factors with RSV cases

In the second monsoon season, we only observed significant positive correlation between temperature and RSV infections.

Meteorological factors	Range	Mean±S.d	Correlation coefficient[a]	p-value	B-value[b]	p-value
Rainfall (mm)	104-678	346.1±133.8	-0.017	0.515	-0.000	0.516
Rainy Days (n)	11-29	19.4±4.4	-0.038	0.140	-0.003	0.103
Temperature (°C)	26.7-29.4	28.3±0.6	0.069	0.007**	0.033	0.013*
Relative Humidity (%)	62.9-82.0	72.9±4.3	-0.044	0.083	-0.005	0.027

[a] indicates results from Spearman's rank correlation
[b] indicates results from multiple bivariate logistic regression
*significance level at $p<0.05$
** significance level at $p<0.01$

Supported by logistic regression, we observe that temperature is positively associated with RSV infections while others have no significance. Generally, we observe that the second monsoon season has higher amount of rainfalls and rainy days. Average temperature during this season is also smaller than the first, which possibly explained its significance to RSV transmission due to the drastic change in temperature. Meanwhile, the second monsoon season has relatively stable weather conditions throughout, with average rainy days in two-thirds of a month. Thus, this may explain the less significant relationship of rainfalls and rainy days to RSV infection.

Supplementary analysis 3: The effects of Covid-19

(a) 2017-2019 (pre-covid)

Table S4. Characteristics of pre-covid study population (2017-2019)

Indicator (N=1988)	RSV Positive, n(%)	RSV Negative, n (%)
Nasopharyngeal swab result	270 (15.7)	1718 (84.3)
Age (years)		
<6 months	84 (19.9)	338 (80.1)
6 months to 2 years	149 (15.6)	806 (84.4)

2.1 years to 5 years	33 (6.8)	452 (93.2)
>6 years	4 (3.2)	122 (96.8)
Years: n(%)		
2017	47 (8.9)	478 (91.1)
2018	56 (10.8)	463 (89.2)
2019	167 (17.7)	777 (82.3)
Ethnicity n (%)		
Malay	245 (13.9)	1507 (86.1)
Chinese	16 (12.7)	110 (87.3)
Indian	3 (10.3)	26 (89.7)
Others	6 (7.4)	75 (92.6)

Figure S1 (a–d). Average monthly distribution of RSV positive cases and various meteorological factors over the period of 3 years (2017-2019)

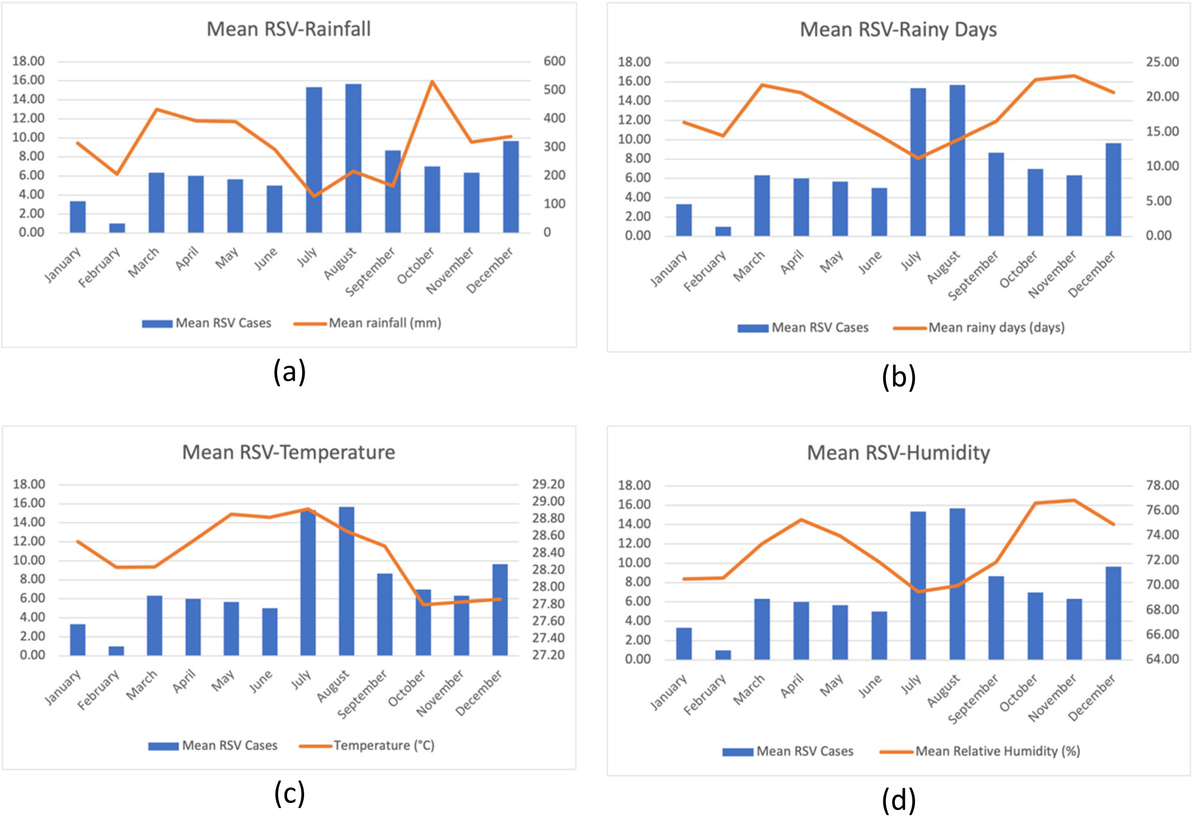

Table S5. Correlation of meteorological factors with RSV cases during pre-covid (2017-2019)

Meteorological factors	Range	Mean±S.d	Correlation coefficient[a]	p-value	B-value[b]	p-value
Rainfall (mm)	72-678	307.3±146.0	-0.054	0.015*	-0.001	0.007**
Rainy Days (n)	9-29	17.7±5.1	-0.072	0.001**	-0.005	0.001**
Temperature (°C)	26.7-29.4	28.4±0.7	0.035	0.117	0.023	0.043*
Relative Humidity (%)	64.7-82.3	72.9±3.9	-0.069	0.002**	-0.006	0.003**

[a] indicates results from Spearman's rank correlation
[b] indicates results from multiple bivariate logistic regression
*significance level at $p<0.05$
** significance level at $p<0.01$

(b) 2020-2021 (During Covid)

Table S6. Characteristics of post-covid study population (2020-2021)

Indicator (N=953)	RSV Positive, n(%)	RSV Negative, n (%)
Nasopharyngeal swab result	165 (17.3)	788 (82.7)
Age (years)		
<6 months	33 (20.7)	126 (79.3)
6 months to 2 years	85 (24.5)	262 (76.5)
2.1 years to 5 years	27 (8.3)	300 (91.7)
>6 years	20 (16.7)	100 (83.3)
Years: n(%)		
2020	80 (15.0)	453 (85.0)
2021	85 (20.2)	335 (79.8)
Ethnicity n (%)		
Malay	157 (18.4)	697 (81.6)
Chinese	6 (12.5)	42 (87.5)
Indian	0 (0)	15 (100)
Others	2 (5.6)	34 (94.4)

Figure S2 (a–d). Average monthly distribution of RSV positive cases and various meteorological factors over the period of 3 years (2017-2019)

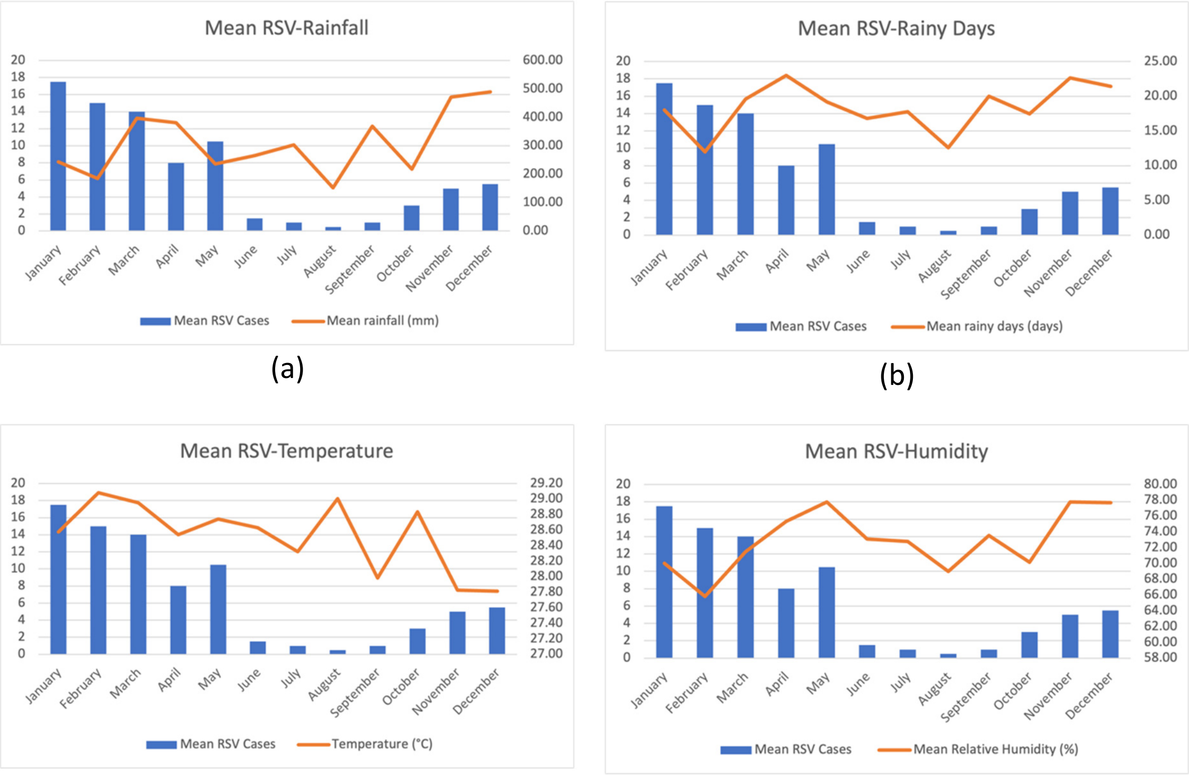

(a) (b) (c) (d)

Table S7. Correlation of meteorological factors with RSV cases during post covid (2020-2021)

Meteorological factors	Range	Mean±S.d	Correlation coefficient[a]	p-value	B-value[b]	p-value
Rainfall (mm)	104-563	311.2±118.1	-0.043	0.187	-0.001	0.197
Rainy Days (n)	11-23	18.5±3.8	-0.004	0.894	-0.002	0.588
Temperature (°C)	27.6-29.3	28.6±0.5	0.037*	0.250	0.034*	0.148
Relative Humidity (%)	62.9-78.8	72.3±4.0	-0.002	0.941	-0.001	0.807

[a] indicates results from Spearman's rank correlation
[b] indicates results from multiple bivariate logistic regression
*significance level at $p<0.05$
